# Euphorbia Kansui Reactivates Latent HIV

**DOI:** 10.1371/journal.pone.0168027

**Published:** 2016-12-15

**Authors:** Daniele C. Cary, Koh Fujinaga, B. Matija Peterlin

**Affiliations:** 1 Department of Medicine, University of California at San Francisco, San Francisco, CA, United States of America; 2 Department of Microbiology, University of California at San Francisco, San Francisco, CA, United States of America; 3 Department of Immunology, University of California at San Francisco, San Francisco, CA, United States of America; Baylor College of Medicine, UNITED STATES

## Abstract

While highly active anti-retroviral therapy has greatly improved the lives of HIV infected individuals, these treatments are unable to eradicate the virus. Current approaches to reactivate the virus have been limited by toxicity, lack of an orally available therapy, and limited responses in primary CD4+ T cells and in clinical trials. The PKC agonist ingenol, purified from *Euphorbia* plants, is a potent T cell activator and reactivates latent HIV. Euphorbia kansui itself has been used for centuries in traditional Chinese medicine to treat ascites, fluid retention, and cancer. We demonstrate that an extract of this plant, Euphorbia kansui, is capable of recapitulating T cell activation induced by the purified ingenol. Indeed, Euphorbia kansui induced expression of the early T cell activation marker CD69 and P-TEFb in a dose-dependent manner. Furthermore, Euphorbia kansui reactivated latent HIV in a CD4+ T cell model of latency and in HIV+ HAART suppressed PBMC. When combined with the other latency reversing agents, the effective dose of Euphorbia kansui required to reactive HIV was reduced 10-fold and resulted in synergistic reactivation of latent HIV. We conclude that Euphorbia Euphorbia kansui reactivates latent HIV and activates CD4+ T cells. When used in combination with a latency reversing agent, the effective dose of Euphorbia kansui is reduced; which suggests its application as a combination strategy to reactivate latent HIV while limiting the toxicity due to global T cell activation. As a natural product, which has been used in traditional medicine for thousands of years, Euphorbia kansui is attractive as a potential treatment strategy, particularly in resource poor countries with limited treatment options. Further clinical testing will be required to determine its safety with current anti-retroviral therapies.

## Introduction

Highly active anti-retroviral therapy (HAART) has changed the face of the HIV/AIDS epidemic, allowing infected individuals to live relatively normal lives [1]. However, they must adhere to life-long drug regimens while continuing to suffer from immunological, neurological, and metabolic co-morbidities associated with HIV infection [2]. Even in individuals with undetectable plasma virema, HIV continues to persist in a latent state, integrated into the host genome and transcriptionally silent [3]. Because this virus is not actively replicating, it escapes elimination by HAART, which targets various proteins expressed throughout the viral replicative cycle [4]. Even a brief interruption in therapy results in rapid rebound in plasma virema due to the presence of latent HIV [5–7]. Although HAART appears to be an elegant solution to the epidemic, it cannot completely eradicate HIV from infected individuals. Problems with drug adherence and availability of effective HAART in socioeconomically challenged areas underlie the continued need to search for HIV cure. Latent reservoirs are the major factor preventing complete elimination of HIV and HIV cure [8]. Strategies to reactivate latent virus on HAART and boost immune responses represent an attractive approach to HIV cure.

HIV uses host transcription machinery for its own replication, and factors which activate CD4+ T cells, such as protein kinase C (PKC) and mitogen-activated protein kinase (MAPK) agonists, also reactivate latent HIV [9–12]. The HIV long terminal repeat (LTR), which acts as the HIV promoter, is highly dependent on positive transcription elongation factor b (P-TEFb) for its activation [13]. PKC agonists induce nuclear translocation of nuclear factor kappa B (NF-κB) and increase cellular levels of cyclin T1 (CycT1) and cyclin dependent kinase 9 (CDK9), components of P-TEFb, which are diminished in resting CD4+ T cells [14]. However, most P-TEFb is sequestered in an inactive complex with 7SK RNA and Hexim 1 (Hex1) [15,16]. Thus, full activation of P-TEFb requires its release from the inactive complex, allowing P-TEFb recruitment to gene promoters, where it mediates transcription elongation. T cell activation induces an increase in cellular P-TEFb followed by transient release from the inactive complex [17,18]. Activation of P-TEFb also results in increased synthesis of Hex1 [19], which returns P-TEFb to its inactive complex. This mechanism limits transcription of other P-TEFb dependent genes, such as inflammatory cytokines [16]. In fact, stimulation of T cells *in vitro* with PKC agonists, such as prostratin and bryostratin, produces little inflammatory cytokines, possibly due to this negative feedback loop [20]. While PKC agonists activate NF-κB and increase expression of P-TEFb, a second signal is required to release most P-TEFb from its inactive complex, thus allowing it to be recruited to gene promoters and activate transcription elongation [21,22].

Any approach to fully eradicate HIV requires reactivation of latent HIV. Histone deacetylase and BET bromodomain inhibitors (HDACi and BETi) were the first compounds tested as latency reversing agents (LRA) [23–27]. The primary mechanism by which HDACi and BETi reactivate HIV is by inducing chromatin stress and releasing P-TEFb from its inactive complex [21,22]. While these LRA are able to release P-TEFb from its inactive complex, they do not affect cellular levels of CycT1 and CDK9 or translocate NF-κB into the nucleus [21,22]. This finding explains why these compounds were effective in cell line models of latency, but failed to reactivate latent HIV in primary CD4+ T cells and in clinical trials [28,29]. Levels of P-TEFb are too low in resting T cells, which limits the effects of HDACi and BETi without additional T cell activation [21,30]. Therefore combination therapy, in which non-toxic doses of a T cell activator and an LRA are used together, is the best approach to reactivate latent HIV [20,31,32].

*Euphorbia* plants are the biologic source of a number of therapeutic ingenols. Ingenol angelate is approved as a safe and effective topical treatment for actinic keratosis [33]. Furthermore, Ingenol B (IngB), a semi-synthetic ingenol, has been safely administered as an oral dose to non-human primates [34]. These ingenols reactivate latent HIV in cell lines and primary T cell models of latency and in cells from HAART suppressed patients [14,32,35,36]. Taken together this evidence has led ingenols to be attractive candidates to reactivate latent HIV. However, potent T cell activation alone as a therapeutic approach still has the potential to result in toxic side effects when administered to patients.

In this study we tested a crude extract from *Euphorbia kansui* as a potential agent to reactivate latent HIV. Euphorbia kansui contains 12 ingenols, as well as other active compounds including: sesquiterpenoids, triterpenoids, and euphols, which may all contribute to the biological activity of Euphorbia kansui [37–39]. Euphorbia kansui has been used for thousands of years for the treatment of fluid retention [40], cancer [41], and acities [42] in traditional Chinese herbal medicine. An oral dose of Euphorbia kansui results in minimal toxicity, mainly gastro-intestinal symptoms, such as diarrhea [43]. In this study we determined that an extract of Euphorbia kansui activates resting CD4+ T cells and induces transcription of latently infected HIV. Furthermore, when used in combination with an HDACi (SAHA) or BETi (JQ1), the effective concentration of Euphorbia kansui is greatly reduced and results in synergistic reactivation of latent HIV at doses which had been administered to humans.

## Results

### Euphorbia kansui activates T cells and HIV in a cell line model of latency

**Fig 1:**
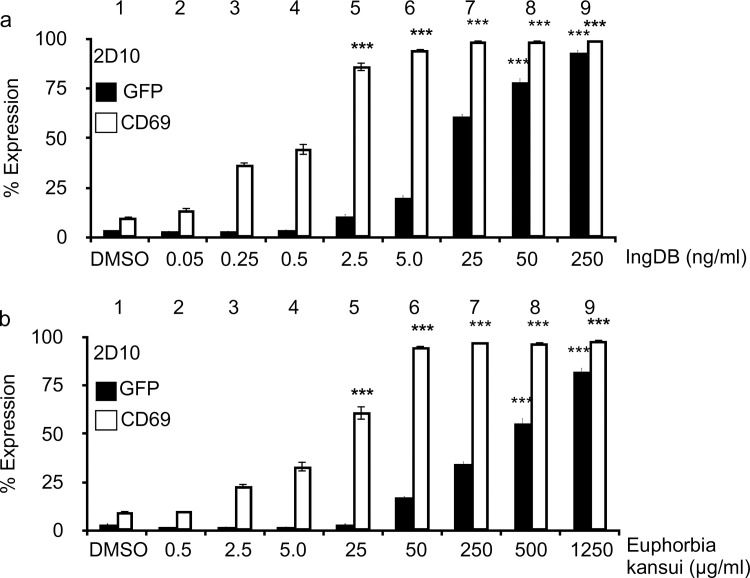
Euphorbia kansui activates T cells and HIV in a cell line model of latency. 2D10 cells were stimulated for 24 hours with DMSO or indicated concentrations of (a) IngDB or (b) Euphorbia kansui (kansui). GFP and CD69 expression were measured by flow cytometry. Triplicate stimulations were performed. Error bars represent standard error of the mean (***p<0.001).

While it is well established that purified ingenol, as well as semi-synthetically modified ingenols, reactivate latent HIV [14,35,36], effects of unpurified Euphorbia kansui on latent HIV are unknown. For this study, an extract of Euphorbia kansui in DMSO was prepared using ground GMP-grade Euphorbia kansui root. This extract was used to activate 2D10 Jurkat T cells which stably express GFP from the HIV LTR [18]. Indeed, ingenol dibenzoate (IngDB) reactivated HIV in 2D10 cells in a dose-dependent manner (Fig 1A) as measured by GFP expression. A 25-fold increase in GFP expression was induced by 24 hour stimulation with 50 ng/ml IngDB (Fig 1A, black bar 8). Euphorbia kansui extract reactivated 2D10 cells (Fig 1B) in a manner comparable to the IngDB positive control (Fig 1A). A 15-fold increase in GFP expression was induced by 500 μg/ml Euphorbia kansui (Fig 1B, black bar 8).

Activation of HIV was measured concurrently with surface expression of CD69, a marker of T cell activation. Ingenols are well established PKC agonists, which activate T cells. Strikingly, concentrations of both IngDB (0.5 ng/ml) (Fig 1A, white bar 4) and Euphorbia kansui (5 μg/ml) (Fig 1B, white bar 5) that are unable to reactivate HIV induced a 10-fold increase in T cell activation.

### Euphorbia kansui activates human primary CD4+ T cells

Latency reversing strategies have historically been successful at reactivating HIV in cell line models of latency [28]. However, cell lines are immortalized and possess abundant levels of NF-κB and P-TEFb. These important cellular transcription factors are limited in resting CD4+ T cells [21,44]. Therefore, it is important to verify that Euphorbia kansui is not just effective in cell lines. Before testing the ability of Euphorbia kansui to reactivate latent HIV in a primary CD4+ model, we wanted to establish that Euphorbia kansui is able to activate primary CD4+ T cells.

**Fig 2:**
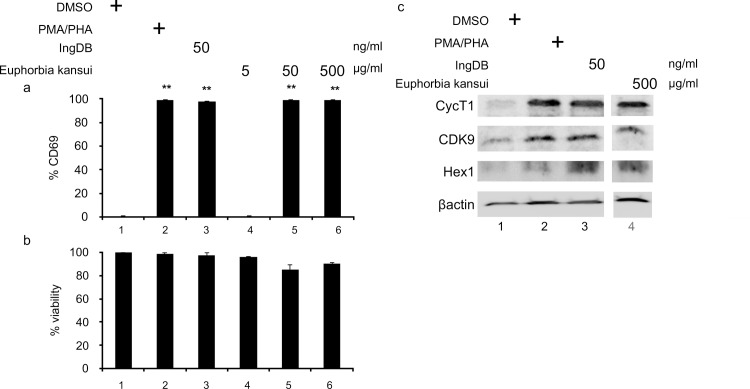
Euphorbia kansui activates human primary CD4+ T cells. (a) Human primary CD4+ T cells were stimulated for 24 hours with DMSO, PMA (2 μg/ml) and PHA (10 ng/ml), IngDB (50 ng/ml) or Euphorbia kansui (5, 50, and 500 μg/ml). CD69 expression was measured by flow cytometry. Percent viability was estimated using the percentage of live lymphocytes in the 10,000 total cells analyzed. Data are representative from stimulations using cells from three healthy donors. Error bars represent standard error of the mean (***p<0.001). (b) Human primary CD4+ T cells were stimulated for 24 hours with DMSO, PMA (10 ng/ml) and PHA (2 μg/ml), IngDB (50 ng/ml) or Euphorbia kansui (5, 50, and 500 μg/ml). Whole cell lystates were separated on 10% SDS PAGE. Membrane was probed for anti-human CycT1, CDK9, Hex1, and β-actin. All samples represented were run on the same gel (space indicates lanes omitted from the figure). Results are representative of western blots of T cells from three healthy donors.

As observed in 2D10 cells, Euphorbia kansui activated T cells at lower concentrations than were required to reactivate HIV (Fig 1B). 50 μg/ml Euphorbia kansui was sufficient to induce a 30-fold increase in CD69 expression, comparable to optimal concentrations of IngDB (50 ng/ml) and PMA (2 μg/ml) /PHA / (10 ng/ml) (Fig 2A, lanes 5, 2, and 3). Importantly, treatment with even the maximum effective concentration of Euphorbia kansui (500 μg/ml) did not result in appreciable reduction in cell viability (Fig 2B, lane 6), as estimated by the percentage of viable lymphocytes from the total cell population.

Resting CD4 + T cells express very low levels of CycT1, an important component of P-TEFb [44]. This deficiency prevents reactivation of resting T cells by HDACi or BETi alone [21,22]. Both CycT1 and CDK9 expression increased in cells treated with 500 μg/ml Euphorbia kansui (Fig 2C, lane 4), similar to the increase observed in cells treated with PMA/PHA and 50 ng/ml IngDB (Fig 2C, lanes 2 and 3). Resting CD4+ T cells also expressed little Hex1, which is bound to CycT1 and CDK9 and sequesters this in a bound inactive complex with 7SK RNA (Fig 2C, lane 1). Hex1 expression increased in cells treated with 500 μg/ml Euphorbia kansui (Fig 2C, lane 4). This increase of Hex1 in Euphorbia kansui treated cells results in the activation induced auto-regulatory shut off of P-TEFb. Indeed, following 24 hour treatment with Euphorbia kansui, we detected no mRNA expression of inflammatory cytokines (data not shown), which is consistent with previously published reports of primary T cell treated with the PKC agonists bryostatin and prostratin *in vitro* [20].

### Euphorbia kansui reactivates HIV in a human CD4+ T cell model of HIV latency

While we observed expression of CD69 in primary CD4+ T cells at 50 μg/ml Euphorbia kansui (Fig 2A, lane 2), this concentration was not necessarily sufficient to reactivate latent HIV in the 2D10 cells (Fig 1B, black bar 6). Thus, for it was important to determine reactivating effects of Euphorbia kansui on latent HIV in a human primary CD4+ T cells.

**Fig 3:**
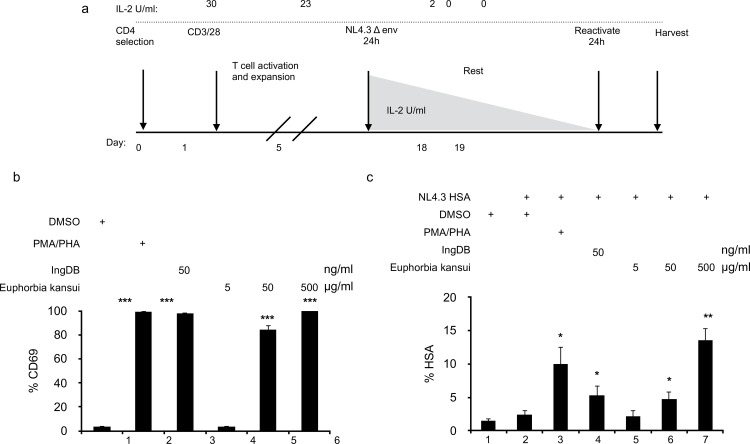
Euphorbia kansui reactivates HIV in a model of HIV latency using resting human CD4+ T cells. (a) Human primary CD4+ T cells were activated and expanded with CD3/CD28 beads and 30 U IL-2/ml for 7 days. Cells were infected with VSV-G-pseudotyped HIV-1 NL4.3 HSA R+E- for 24 hours. Infectious virus was removed and cells were maintained over 12 days with decreasing concentrations of IL-2 to establish latency. Uninfected cells were maintained in the same conditions, for uninfected controls and to determine T cell reactivation. At 12 days post infection, cells were stimulated for 24 hours with DMSO, PMA (2 μg/ml) and PHA (10 ng/ml), IngDB (50 ng/ml) or Euphorbia kansui (5, 50, and 500 μg/ml) (***p<0.001). (b) CD69 expression was measured by flow cytometry. (c) HSA expression was measured by flow cytometry. Data are representative of infections using cells from three healthy donors. Error bars represent standard error of the mean (*p<0.05, **p<0.01).

We utilized our primary CD4+ T cell model of HIV latency (Fig 3A) [14]. Human peripheral blood mononuclear cells (PBMC) were isolated and CD4+ T cells were purified using negative bead selection. CD4+ T cells were activated with CD3/CD28 beads and 30 U/ml IL-2. Cells were activated and expanded over 5 days. Activated T cells were infected through spinoculation with envelope negative HIV-1 NL4.3 expressing murine heat shock antigen (HSA) pseudotyped with VSV-G [45,46]. A replication incompetent virus was used to provide a single round infection and to prevent spreading infection. Cells were infected for 24 hours, after which infectious virus was washed off and cells were given fresh media with 23 U IL-2/ml. Over 12 days, IL-2 concentrations were gradually reduced to 2 U/ml. Uninfected controls were also activated and taken to a resting state over 12 days. At 18 day post-infection, cells were treated with reactivating stimuli for 24 hours.

Following the 12 day course of decreasing IL-2 concentrations, CD4+ T cells treated with DMSO alone did not express CD69 (Fig 3B, lane 1), suggesting that our latency model conditions induced a quiescent state. In agreement with our observations in 2D10 (Fig 1B) and resting CD4+ T cells (Fig 2A), 50 and 500 μg/ml Euphorbia kansui induced a 30-fold increase in CD69 expression (Fig 3B, lanes 5 and 6) equal to optimal concentrations of PMA/PHA and IngDB (Fig 3B, lanes 2 and 3).

Following the 12 day course of decreasing IL-2, infected cells treated with DMSO did not express HSA (Fig 3C, lane 2), confirming that our culture conditions induced a latent state. Reactivation with PMA/PHA resulted in a 4-fold increase in the percentage of HSA expressing cells (Fig 3C, lane 3), while IngDB alone induced a 1.5-fold increase (Fig 3C, lane 4). Euphorbia kansui reactivated latent HIV in a dose-dependent manner, as observed by increasing expression of HSA (Fig 3C, lanes 5–7). At the optimal dose of 500 μg/ml, we observed a 6-fold increase in HSA expression (Fig 3C, lane 7), which was a 4.5-fold increase over IngDB alone. Consistent with 2D10 cells, the suboptimal concentration of 50 μg/ml Euphorbia kansui induced CD69 expression (Fig 3B, lane 5), however in primary CD4+ T cells, 50 μg/ml Euphorbia kansui reactivated HIV similar to the IngDB control (Fig 3C, lane 4 and 5).

### Combination therapy decreases the effective dose of Euphorbia kansui

**Fig 4:**
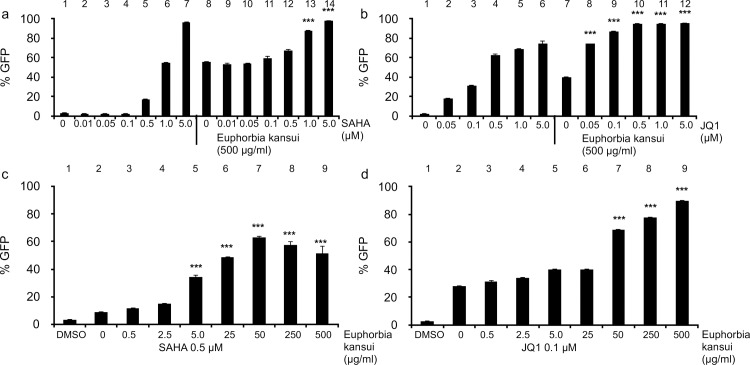
Combination therapy decreases the effective dose of Euphorbia kansui and SAHA or JQ1. 2D10 cells were stimulated for 24 hours with DMSO or (a) indicated concentrations of SAHA alone +/- 500 μg/ml Euphorbia kansui, (b) indicated concentrations of JQ1 alone +/- 500 μg/ml Euphorbia kansui, (c) 0.5 μM SAHA and indicated concentrations of Euphorbia kansui, (d) and 0.1μM JQ1 and indicated concentrations of Euphorbia kansui. GFP expression was measured by flow cytometry. Triplicate stimulations were performed (***p<0.001).

HDACi and BETi fail to reactivate latent HIV in more relevant models and clinical trials, however when paired with a PKC agonist, both reagents are effective in primary CD4+ T cells and patient samples [20,31,32]. We chose the two best characterized LRA, an HDACi (SAHA) and a BETi (JQ1), to test in combination with Euphorbia kansui [22,25]. Using the optimal concentration of Euphorbia kansui (500 μg/ml) decreasing concentrations of SAHA and JQ1 were tested to determine the concentration required to maximally activate 2D10 cells. 5 μM SAHA (Fig 4A, lane 7) and 0.5 μM JQ1 (Fig 4B, lane 4) alone were required to achieve maximum GFP expression in 2D10 cells. However, 1.0 μM SAHA (Fig 4A, lane 13) and 0.05 μM JQ1 (Fig 4B, lane 8) in combination with 500 μg/ml Euphorbia kansui resulted in an increase over Euphorbia kansui alone.

Next, using reduced concentrations of SAHA and JQ1, the concentration of Euphorbia kansui was titrated down. While 0.5 μM SAHA and 500 μg/ml Euphorbia kansui resulted in only a modest increase in 2D10 reactivation (Fig 4A, lane 12), this concentration was sufficient to increase the effects of lower concentrations of Euphorbia kansui (Fig 4C, lane 5–7). At 0.5 μM SAHA, concentrations as low at 5 μg/ml Euphorbia kansui (Fig 4C, lane 5) reactivated HIV in 2D10 cells. Maximum reactivation was observed at 50 μg/ml Euphorbia kansui (Fig 4C, lane 7). While, 0.05 μM JQ1 resulted in an increase in HIV reactivation with 500 μg/ml Euphorbia kansui, there was no strong effect in combination with lower doses of Euphorbia kansui (data not shown), therefore, 0.1 μM JQ1 was used for the Euphorbia kansui titration (Fig 4D). At 0.1 μM JQ1, 50 μg/ml Euphorbia kansui was sufficient to reactivate HIV in 2D10 cells (Fig 4D, lane 7). Based on the Bliss independence model for HIV drug combinations, as previously described by Laird et. al [20], both 0.5 μM SAHA and 0.1 μM JQ1 in combination with 50 μg/ml Euphorbia kansui exhibited synergistic reactivation of HIV in 2D10 cells.

### Combination therapy activates T cells and reactivates HIV in a human CD4+ cell model of HIV latency

**Fig 5:**
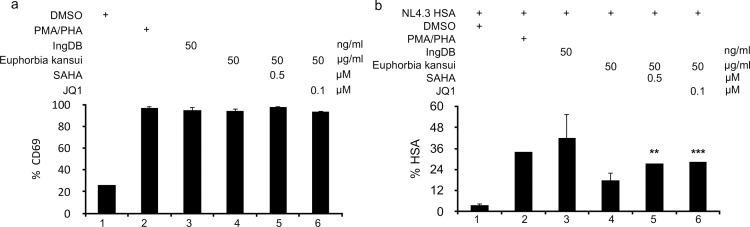
Combination therapy of Euphorbia kansui and SAHA or JQ1 reactivates latent HIV and activates T cells. Human primary CD4+ T cells were activated and expanded with CD3/CD28 beads and 30 U/ml IL-2/ml for 7 days. Cells were infected with VSV-G-pseudotyped NL4.3 HSA R+E- for 24 hours. Infectious virus was removed and cells were maintained over 12 days with decreasing concentrations of IL-2 to establish latency. Uninfected cells were maintained in the same conditions, for uninfected controls and to determine T cell reactivation. At 12 days post infection, cells were stimulated for 24 hours with DMSO, PMA (2 μg/ml) and PHA (10 ng/ml), IngDB (50 ng/ml), Euphorbia kansui (50 μg/ml), Euphorbia kansui (50 μg/ml) and SAHA (0.5 μM), or Euphorbia kansui (50 μg/ml) and JQ1 (0.1μM). (a) CD69 expression was measured by flow cytometry. (b) HSA expression was measured by flow cytometry. Data are representative of 3 healthy donors (**p<0.01, ***p<0.001).

Using our primary CD4+ latency model (Fig 3A), quiescent CD4+ T cells were stimulated with suboptimal concentrations of Euphorbia kansui (50 μg/ml) and SAHA (0.5 μM) or JQ1 (0.1 μM). All treatments induced CD69 expression to the same level as the PMA/ PHA positive control (Fig 5A). There was no observed difference in CD69 expression between Euphorbia kansui alone or in combination with SAHA or JQ1 (Fig 5A, lanes 4–6). Optimal concentrations of both PMA/PHA and IngDB reactivated HIV from quiescent CD4+ T cells (7.5- and 9.5-fold respectively) (Fig 5B, lanes 2 and 3). The suboptimal concentration of 50 μg/ml Euphorbia kansui induced a 3.5-fold increase in HSA expressing cells, while combination treatment of SAHA and JQ1 with Euphorbia kansui induced a 6-fold increase in HSA expressing cells (Fig 5B, lanes 5 and 6).

### Combination therapy reactivates HIV in PBMC from HIV+ HAART suppressed patients

**Fig 6:**
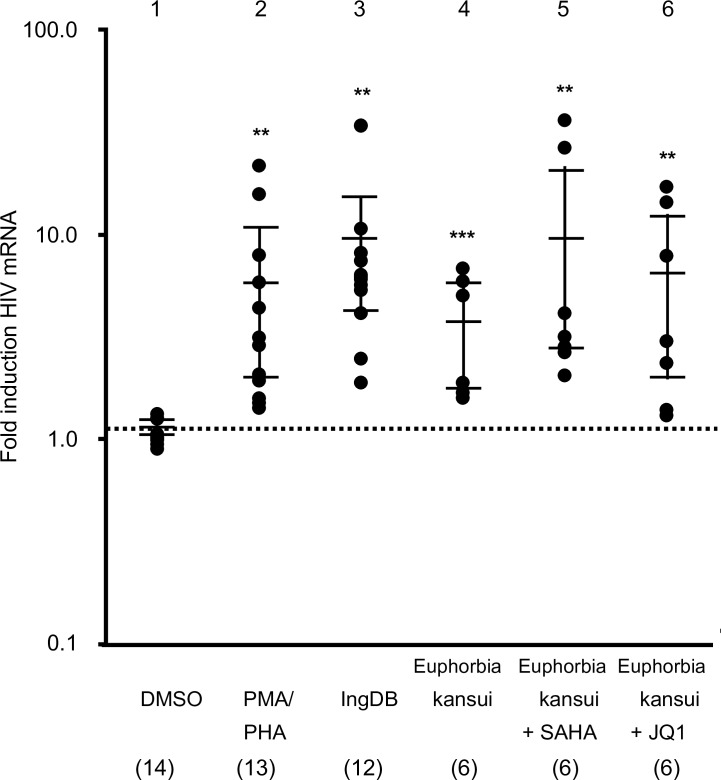
Combination therapy of Euphorbia kansui and SAHA or JQ1 reactivates HIV in PBMC from HIV+ HAART suppressed patients. PBMC were isolated from whole blood samples from HIV+ HAART suppressed patients (Table 1), and stimulated for 24 hours with DMSO, PMA (2 μg/ml) and PHA (10 ng/ml), IngDB (50 ng/ml), Euphorbia kansui (50 μg/ml), Euphorbia kansui (50 μg/ml) and SAHA (0.5 μM), or Euphorbia kansui (50 μg/ml) and JQ1 (0.1μM). Numbers in parentheses indicate number of individuals used for each treatment. Reactivation of latent HIV was measured by real time RT-PCR analysis of HIV Gag/Pol transcripts over DMSO control. Fold change over DMSO control is presented on a log scale. Bars represent the median and interquartile range of each treatment (**p<0.01, ***p<0.001).

Finally, we tested combinations of Euphorbia kansui and SAHA or JQ1 on HIV+ HAART suppressed patient samples (Table 1). Patient PBMC were treated with PMA/PHA, IngDB, and Euphorbia kansui alone or in combination with SAHA or JQ1. Patient PBMC treated with PMA/PHA had a median 5-fold increase in expression of HIV mRNA compared to the DMSO control (Fig 6, lane 1). The supoptimal dose of 50 μg/ml Euphorbia kansui induced a median 3-fold increase in HIV expression (Fig 6, lane 4), while Euphorbia kansui and SAHA induced a 9-fold increase in HIV expression (Fig 6, lane 5), and Euphorbia kansui and JQ1 induced a 5-fold increase in HIV expression (Fig 6, lane 6).

**Table 1:** Characteristics of HIV-1–infected HAART suppressed patients.

Patient	Age	Sex	Viral load ^a^	CD4^+^ T cell count ^b^	ART regimen	Time on HAART ^c^
1	30	M	<40	706	EGV/TDF/FTC/COB	2.1
2	58	M	<40	647	EFV/TDF/FTC	8.3
3	63	M	<40	585	FTC/TDF,ATV,RTV	16.0
4	59	M	<40	633	EFV/TDF/FTC, RGV	8.6
5	56	F	<40	942	FTC/TDF, LPV/r	6.5
6	48	M	<40	767	FTC/TDF, NVP	6.3
7	31	F	<40	781	EGV/TDF/FTC/COB	0.7
8	64	M	<40	833	FTC/TDF, ETV	11
9	68	M	<40	917	EFV/TDF/FTC	9.4
10	43	M	<40	405	FTC/TDF,RGV	4.1
11	61	M	<40	747	TCV, RPV	15.4
12	53	M	N/A ^d^	623	ABC/3TC,RTV,DRV,RGV	12.4
13	68	M	<40	949	EFV/TDF/FTC	8.9
14	68	M	<40	360	3TC, NVP, RTV, DRV, RGV	19.3

Abbreviations: M, male; F, female; 3TC, Iamivudine; ABC, Abacavir; ATV, Atazanavir; DRV, Darunavir; COB, Cobicistat; EFV, Efavirenz; EGV, Elvitegravir; ETV, Etravirine; FTC, Emtricitabine; LPV/r, Lopinavir (w/ritonavir);NVP, Nevirapine; RGV, Raltegravir; RPV, Rilpivirine, RTV, Ritonavir; TCV, Dolutegravir; TDF, Tenofovir

^a^ Plasma viral load (copies/ml)

^b^ Peripheral CD4 (cells/ml)

^c^ Time under ART (years)

^d^ No recent viral loads available in SCOPE. Long-term suppressed per patient self report.

## Discussion

In this study we determined that a crude preparation of *Euphorbia Euphorbia kansui* reactivates latent HIV in 2D10 cells, primary CD4+ T cells, and HAART suppressed patient samples. Using the 2D10 cell model of latency, we established that Euphorbia kansui reactivates HIV in a dose-dependent manner, similar to purified IngDB. Euphorbia kansui also activated CD4+ T cells, leading to increased cellular expression of P-TEFb. Utilizing our CD4+ T cell model of latency, we determined that Euphorbia kansui reactivated latent HIV in quiescent T cells. Most importantly, Euphorbia kansui reactivated HIV in HIV+ HAART suppressed patient PMBC. Because Euphorbia kansui activated T cells at lower concentrations than those required to reactivate HIV, we hypothesized that a combination therapy, with agents that require T cell activation and cellular P-TEFb, would reduce effective concentrations of both agents. Indeed, we found that Euphorbia kansui and SAHA or JQ1 resulted in synergistic reactivation of latent HIV at suboptimal concentrations. This combination therapy also reactivated latent HIV in CD4+ T cells and in HAART suppressed patients. Taken together, these results demonstrate that Euphorbia kansui is an excellent PKC agonist to reactivate latent HIV, which could be used in combination with another LRA.

Batch to batch variation is a concern when studying a natural product. We have prepared several extractions of Euphorbia kansui, subjected them to repeated freeze/thaw cycles, and did not observe any subsequent loss of efficacy. This finding demonstrates the stability and consistency between Euphorbia kansui preparations. The dose response in 2D10 cells provides an easy method to test different preparations of Euphorbia kansui, and can be used to determine batch to batch variation between different Euphorbia kansui root preparations or other *Euphorbia* plants. While the exact components of each preparation used in this study were not determined by HPLC, the active compounds in Euphorbia kansui have been analyzed extensively elsewhere [37–39]. Euphorbia kansui contains 12 ingenols, as well as sesquiterpenoids, triterpenoids, and euphols [37–39]. This combination of active compounds could contribute to the observed reactivation of latent HIV, and the reported anti-inflammatory properties of euphols [47,48] may mitigate the adverse inflammatory effects of ingenols in Euphorbia kansui. The efficacy of Euphorbia kansui is equivalent to that of the purified IngDB, and a titration of Euphorbia kansui in 2D10 cells demonstrated dose-dependent responses of T cell activation and HIV reactivation.

In this study we observed reactivation of HIV from HIV+ HAART suppressed patient samples. However we did not observe robust reactivation in all samples treated. Individuals on effective HAART have levels of plasma viremia below the limits of detection. It is estimated that the size of the latent reservoir is between 10^6^ to 10^8^ latently infected cells [8]. Given that samples in this study come from individuals on long term therapy, it is possible that the samples we received contain few or no latently infected cells or defective HIV [3,49]. In fact, the most potent stimulation used, PMA/PHA also failed to reactivate HIV in these four samples.

When considering potential therapeutic agents, toxicity and safety are crucial factors. IngB, synthetically modified from ingenol, has been safely tested in macaques at a concentration of 400 μg/kg/day [34]. IngB is approximately 200-fold less effective at activating latent HIV in 2D10 cells than IngDB [14], therefore an approximate safe therapeutic dose of IngDB would be 2 μg/kg/day. There is a 10,000-fold difference in the efficacy between IngDB and Euphorbia kansui: IngDB reactivated 2D10 at 5 ng/ml (Fig 1A), while 50 μg/ml Euphorbia kansui was required. Using the effective dose in macaques, the differential efficacy between IngB and IngDB, and the 10,000-fold difference in effective concentration of Euphorbia kansui and IngDB, we estimate the effective dose of Euphorbia kansui to be ~ 20 mg/kg/day. This dose falls within the 1.5–8 g/50 kg person/day Euphorbia kansui dose used in traditional Chinese herbal medicine [43], demonstrating that concentrations effective at reactivating HIV *in vitro* are within the dose range previously reported to be safe for use in humans. Nevertheless, rigorous clinical testing is required to determine its safety and efficacy in HIV+ anti-retroviral treated individuals.

The combination therapy of Euphorbia kansui with SAHA or JQ1 provides a more effective approach than using either a PKC agonist or LRA alone [20,31,32]. Using these two LRA we observed synergistic responses with Euphorbia kansui. Combination therapy not only reduced the effective dose of Euphorbia kansui ten-fold, but also decreased the concentrations of SAHA and JQ1. SAHA and JQ1 release P-TEFb from its inactive complex, thereby allowing P-TEFb to activate HIV transcription [21,22]. However, diminished levels of P-TEFb in resting T cells limit reactivation by SAHA and JQ1 in primary cell systems or clinical applications [44]. Treatment with Euphorbia kansui activates CD4+ T cells and increases cellular expression of P-TEFb. Furthermore, activation of T cells by Euphorbia kansui increases cellular expression of Hex1, mediating the return of P-TEFb to its inactive complex [19]. This autoregulatory negative feedback limits activation by P-TEFb responsive genes such as inflammatory cytokines, which may explain a lack of inflammatory cytokines observed in T cells treated with PKC agonists *in vitro* [20]. While other P-TEFb responsive genes will be turned off by Hex1 increases, HIV transcription is less affected. Following initial rounds of HIV transcription, HIV Tat is produced. By competing with Hex1 for binding to 7SK RNA, Tat liberates P-TEFb from its inactive complex and drives subsequent rounds of HIV transcription [15]. Finally, because of the non-redundant and complementary mechanisms of Euphorbia kansui and HDACi or BETi, these therapies can potentially be staggered to help ameliorate patient discomfort. As a combination therapy, lower concentrations of both compounds can be administered, potentially limiting toxic side effects of either.

Euphorbia kansui is a practical approach for using a PKC agonist to reactivate latent HIV. Euphorbia kansui has a relatively rapid response when used in traditional Chinese medicine for ascites, resulting in loss of water retention within 2 hours of administration [43]. These gastro-intestinal symptoms can also be used as an indicator of bioactivity in a clinical setting. These side effects are reduced by administering Euphorbia kansui with jujube fruit [43]. Euphorbia kansui is proposed as an oral therapy, which greatly increases the ease at which it can be administered. Purified ingenol is currently only available as a topical treatment [33], and prostratin is administered as an injection [50]. *Euphorbia* species grow ubiquitously world-wide [38]. Bryostatin is derived from marine bryozoans, Bugula neritina. Approximately one ton of bryozoans are required to synthesize a gram of bryostatin, making it impractical and expensive as a widespread therapeutic option [51]. Currently a number of less toxic prostratin and bryostain synthetic analogues, as well as IngB, are under investigation; however these remain to be tested in humans [14,52,53].

Euphorbia kansui is a fairly ubiquitous plant, making it a useful therapeutic in areas without access to expensive treatment options. Euphorbia kansui, as an unpurified preparation, has been used in traditional Chinese medicine for thousands of years [43], and Aveloz, or Euphorbia tirucalli, has been used in Brazil to treat cancer [41]. Euphol, another bioactive component of Euphorbia Euphorbia kansui has anti-inflammatory properties [47,48]. The natural combination ingenols and euphols in Euphorbia kansui may prove a safer therapeutic approach than treatment with purified ingenol alone, as the anti-inflammatory properties of the euphol potentially reduce the inflammatory side-effects of the ingenols. Euphorbia kansui would be a useful treatment option in sub-Saharan Africa, which accounts for the largest population of HIV infected individuals world-wide. Combining eastern and western therapies may seem unconventional; however this is becoming a commonly used means to enhance western medical strategies [40]. Furthermore, there is a precedence of plant based compounds leading to conventional medical treatments including quinine, aspirin, cyclosporine A, and taxol [54,55]. The discovery of artemisinin, an anti-malarial treatment isolated from the traditional Chinese medicinal herb qinghaosu was recently recognized with a Nobel prize in Medicine [56]. Two recent studies have highlighted procyandin, which was isolated from traditional medicinal plants. *Theobroma cacao*, the plant source of cocoa, has cardiovascular and metabolic health benefits as well as potential anti-inflammatory and anti-cancer properties. Procyanidin from cacao reactivates latent HIV via the MAPK pathway, and shows synergistic activation when added in combination with PMA [12]. A second procyanidin compound isolated from *Polygonum cuspidatum Sieb*. *et Zucc*, procyanidin C-13,3',3"-tri-O-gallate, also reactivates latent HIV in cell line models [57]. When taken together with our results using Euphorbia kansui, there is an emerging precedence for exploring nonpharmacological approaches to treat HIV. These studies, using the same established and scientifically rigorous bioassays used to validate pharmacological drug compounds, should help modify perception of natural plant based approaches to target latent HIV. Implementing more affordable plant based approaches into current HIV therapies is especially vital to efforts in resource limited settings affected by HIV/AIDS.

## Materials and Methods

### Cell lines and primary CD4+ T cells

2D10 cells (obtained from Dr. Jonathan Karn at Case Western Reserve University) are a Jurkat based HIV latency cell line model that contains attenuated Tat and d2EGFP in the place of Nef [18]. Reactivation of latent HIV in 2D10 cells was measured by d2EGFP expression by flow cytometry. Trima residuals from healthy donors, from Trima aphoresis collection and enriched for PBMC, were obtained from Blood Center of the Pacific (San Francisco, CA). CD4+ T cells were selected from bulk PBMC using negative bead selection (Dynal CD4+ untouched beads, Invitrogen). Primary CD4+ T cells were activated and expanded using CD3/CD28 beads (Invitrogen) and 30 U/ml interleukin 2 (IL-2) for 5 days.

### HIV+ HAART suppressed patient samples

Bulk PBMC were isolated from 14 whole blood samples obtained from the University of California, San Francisco SCOPE cohort from subjects on Food and Drug Administration-approved anti-retroviral agents with plasma HIV RNA levels below the limit of detection at the day of sample collection (Table 1). Sample collection and patient information was handled solely by the personnel at Zuckerberg San Francisco General Hospital, and the authors had no access to patient identification. Samples obtained were not archived samples.

Written informed consent was obtained from the participant before sample collection occurred. The research participant was given a copy of the signed informed consent document, and a signed copy of the informed consent is retained in Zuckerberg San Francisco General Hospital’s medical records. Only staff of the SCOPE cohort or physician investigators listed on the protocol obtain informed consent. This consent procedure was approved by the University of California, San Francisco Human Research Protection Program Committee on Human Research.

### Euphorbia kansui extract

Powdered Euphorbia kansui root was obtained from Baoji F.S. Biological Development Co. Ltd. (Shaanxi, China). Extract was prepared by mixing 0.5 g ground Euphorbia kansui root powder/1ml DMSO for 1 hr on a bi-directional mixer followed by filtration through a 0.45 μm filter. Extracts were stored at -80°C for long term storage, and working stocks were stored at -20°C.

### Cell culture and reactivation conditions

Cells were maintained in RPMI 1640 supplemented with 10% FBS and Penicillin/Streptomycin at 37°C with 5% CO_2_. Cells were stimulated at a concentration of 1 x 10^6^ cells/ml with DMSO (1 μl/ml), Phorbol myristate acetate (PMA) (Sigma Aldrich) (2 μg/ml) and Phytohaemagglutinin (PHA) (Sigma Aldrich) (10 ng/ml), or indicated concentrations of Ingenol Dibenzoate(Sigma Aldrich), Euphorbia kansui extract, SAHA (Martin Delaney Collaboratory of AIDS Researchers for Eradication (CARE)), or JQ1 (CARE). Cells were seeded in triplicate wells and stimulated for 24 hours. Approximately 5–10 x 10^6^ cells/5-10 ml on 10 cm plates were stimulated to obtain lysates for protein expression.

### Generation of HIV-1 infectious titers and infections

Infectious stocks of HIV-1 were generated by transfecting 293T cells with 15 μg of pNL4.3-Nef(+)-HSA (National Institutes of Health AIDS Research and Reference Reagent Program, Division of AIDS, NIAID, NIH: pNL4-3.HSA.R+.E- from Dr. Nathaniel Landau [45,46]) and 3 μg of VSV-G using calcium phosphate. Viral supernatants were harvested and filtered through a 0.45 μm disc at 48 hours post transfection. Approximately 0.5 x 10^6^ pg p24 of infectious virus were added per 1 x 10^6^ activated CD4+ T cells. Cells were spinoculated for 90 min at 2000 rpm with polybrene (2 μg/ml). 24 hours post infection, cells were washed twice to thoroughly remove initial infectious virus.

### Human CD4+ T cells model of HIV latency

Following 5 day activation and expansion with CD3/28 beads and 30 U/ml IL-2, CD3/28 beads were removed from culture. Approximately 60 x 10^6^ CD4+ T cells were spinoculated with HIV-1 NL4.3 HSA. Cells were cultured 24hours in infectious virus with 23 U/ml IL-2. After 24 hour infections, cells were washed 2 x in PBS to remove infectious viral supernatants and returned to culture at a density of 2 x 10^6^ cells/ ml with 23 U/ml IL-2. 30 x 10^6^ CD4+ T cells were cultured as uninfected controls. IL-2 concentrations were reduced over the course of 12 days to induce cell quiescence and HIV latency (16 U/ml on day 3, 9 U/ml on day 6, 2 U/ml on day 9). Latently infected and uninfected control cells were reactivated with DMSO or appropriate compounds for 24 hours and samples were collected.

### Flow cytometry

Cells were harvested 24 hours post reactivation and washed in cold PBS and 0.5 x 10^6^ cells were allotted to each tube. Cells were stained with PE mouse anti-human CD69 (BD Biosciences), FITC rat anti-mouse HSA (BD Biosciences), or fixed immediately to analyze GFP expression. Cells were fixed in 2% paraformaldehyde and analyzed using the BD Biosciences FACScaliber and CellQuest Pro software at the UCSF Parnassus Flow Cytometry Core. Cells were gated on the live lymphocyte gate using the forward and side scatter plot, and the percentage of live lymphocytes in 10,000 collected total cells was used as an estimate for cell viability.

### Western blot analysis of protein expression

Whole cell lysates were generated using lamemmli buffer (Bio-rad) in the presence of proteinase inhibitor cocktail. Lysates were run on 10% SDS-PAGE and transferred onto a nitrocellulose membrane. Membranes were cut at approximately 60 kDa; the top portion of the membrane was used to probe for cyclin T1 (CycT1) (75 kDa), the bottom portion was used to probe for CDK9 (40 kDa), Hexim 1 (Hex1) (54 kDa) and βactin (55 kDa). Membranes were blocked in 5% non-fat milk (NFM) for at least 1 hour and blotted overnight with rabbit anti-human cyclin T1 antibody (Santa Cruz Biotechnology), rabbit anti-human CDK9 (Santa Cruz Biotechnology), rabbit anti-human Hex1 (Santa Cruz Biotechnology), and rabbit anti-human β actin (abcam) in 5% NFM. Membranes were washed 3 x in PBS with 0.05% Tween 20, and then blotted for 1 hour with HRP anti-rabbit IgG, in 5% NFM. After washing 3 x with PBS with 0.05% Tween 20; membranes were treated with ECL Plus chemiluminescence reagent (Promega) for 5 minutes and imaged using Odyssey Fc imaging system and Image Studio software (LI-COR). Reprobed membranes were stripped with NewBlot Stripping Buffer (LI-COR) then washed 3 x with PBS.

### Real time RT-PCR

Total RNA was isolated using Trizol (Life Technologies), and cDNA was amplified using Superscript Reverse Transcriptase III (ThermoFisher). Real time RT-PCR was performed using SensiFAST SYBR Lo-ROX kit (Bioline) and analyzed using Stratagene Mx3005p and MxPro QPCR software. Triplicates were performed for each primer pair and sample. Relative expression was calculated using the delta-delta CT (2-ΔΔct) method [58], using GAPDH as an internal control and DMSO conditions as the calibrator. Data are presented as a fold change over these control values for each patient. Gag/Pol primers used in this study [59]: P1 (forward): TTCT TCAGAGCAGACCAGAGC, P2 (reverse): GCTGCCAAAGAGTGATCTGA. GAPDH primers used in this study: Sense: TCAAGTGGGGCGATGCTGGC, Antisense: TGGGGGCATCAGCAGAGGGG.

### Calculation of drug synergy

To determine whether combination treatment resulted in synergistic activation of HIV in 2D10 cells we used the Bliss independence model previously described. The equation fa_xy,p_ = fa_x_ + fa_y_- (fa_x_)(fa_y_), wherein fa_x,p_ is the prediction based on the individual effects of Euphorbia kansui (fa_x_), SAHA or JQ1 (fa_y_). The individual effects were calculated by the equation fa_x/y/xy_ = (% GFP positive treatment—% GFP positive DMSO)/ (% GFP positive IngDB 50 ng/ml—% GFP positive DMSO). The difference between the observed and predicted values (Δfa_xy_ = fa_xy,o_—fa_xy,p_) was calculated. A value greater than 0 signified synergy, a value equal to 0 indicated Bliss independence, and a value less than 0 indicated antagonism.

### Statistical analysis

Statistical analysis was performed using a Student *t* test, two-tailed distribution, and assuming equal variances.
